# Retraction: The Role of Heat Shock Proteins in Cellular Homeostasis and Cell Survival

**DOI:** 10.7759/cureus.r36

**Published:** 2021-11-18

**Authors:** Abdullah Farhan Y Almalki, Maria Arabdin, Adnan Khan

**Affiliations:** 1 Pathology, University of Malta, Msida, MLT; 2 Pathology, Rehman Medical College, Peshawar, PAK; 3 Pediatrics, Rehman Medical Institute, Peshawar, PAK

It has come to our attention that figures 1 and 3 in this article have been reproduced by the authors without first obtaining permission from the original publishers or authors:

Figure 1: Akerfelt M, Morimoto RI, Sistonen L: https://dx.doi.org/10.1038/nrm2938?utm_medium=email&utm_source=transaction Heat shock factors: integrators of cell stress, development and lifespan. Nat Rev Mol Cell Biol. 2010, 11:545-55. https://dx.doi.org/10.1038/nrm2938 10.1038/nrm2938

 

Figure 3: Pockley AG. https://www.cambridge.org/core/journals/expert-reviews-in-molecular-medicine/article/abs/heat-shock-proteins-in-health-and-disease-therapeutic-targets-or-therapeutic-agents/21095BFDB742E7876E4D1FDBF33E971C Heat shock proteins in health and disease: therapeutic targets or therapeutic agents? Expert Rev Mol Med. 2001, 3:1-21. https://dx.doi.org/10.1017/S1462399401003556 10.1017/S1462399401003556.

In addition, Figure 3 was also not properly attributed to the original source. This was later fixed via correction. After confirming with the publisher of Figure 1 that permission to republish was not obtained, and receiving no response from the authors despite multiple attempts, we have made the decision to retract this article and completely remove Figures 1 and 3 from the online and PDF versions of the article.

